# Exposure of Egyptian Rousette Bats (*Rousettus aegyptiacus*) and a Little Free-Tailed Bat (*Chaerephon pumilus*) to Alphaviruses in Uganda

**DOI:** 10.3390/diseases10040121

**Published:** 2022-12-05

**Authors:** Rebekah C. Kading, Erin M. Borland, Eric C. Mossel, Teddy Nakayiki, Betty Nalikka, Jeremy P. Ledermann, Mary B. Crabtree, Nicholas A. Panella, Luke Nyakarahuka, Amy T. Gilbert, Julian C. Kerbis-Peterhans, Jonathan S. Towner, Brian R. Amman, Tara K. Sealy, Barry R. Miller, Julius J. Lutwama, Robert M. Kityo, Ann M. Powers

**Affiliations:** 1 Department of Microbiology, Immunology and Pathology, Colorado State University, Fort Collins, CO 80523, USA; 2 Arbovirus Diseases Branch, Division of Vector-Borne Diseases, U.S. Centers for Disease Control and Prevention, Fort Collins, CO 80521, USA; 3 Department of Arbovirology, Emerging, and Re-Emerging Infections, Uganda Virus Research Institute, Entebbe, Uganda; 4 Department of Zoology, Entomology, and Fisheries Science, Makerere University, Kampala, Uganda; 5 Animal Plant Health Inspection Service, National Wildlife Research Center, United States Department of Agriculture, Fort Collins, CO 80521, USA; 6 Poxvirus and Rabies Branch, Division of High-Consequence Pathogens, United States Centers for Disease Control and Prevention, Atlanta, GA 30333, USA; 7 Negaunee Integrative Research Center, Field Museum of Natural History, College of Arts & Sciences, Roosevelt University, Chicago, IL 60605, USA; 8 Viral Special Pathogens Branch, Division of High-Consequence Pathogens, United States Centers for Disease Control and Prevention, Atlanta, GA 30333, USA

**Keywords:** chiroptera, surveillance, arbovirus, mosquito, emerging virus, serology, chikungunya, o’nyong-nyong

## Abstract

The reservoir for zoonotic o’nyong-nyong virus (ONNV) has remained unknown since this virus was first recognized in Uganda in 1959. Building on existing evidence for mosquito blood-feeding on various frugivorous bat species in Uganda, and seroprevalence for arboviruses among bats in Uganda, we sought to assess if serum samples collected from bats in Uganda demonstrated evidence of exposure to ONNV or the closely related zoonotic chikungunya virus (CHIKV). In total, 652 serum samples collected from six bat species were tested by plaque reduction neutralization test (PRNT) for neutralizing antibodies against ONNV and CHIKV. Forty out of 303 (13.2%) Egyptian rousettes from Maramagambo Forest and 1/13 (8%) little free-tailed bats from Banga Nakiwogo, Entebbe contained neutralizing antibodies against ONNV. In addition, 2/303 (0.7%) of these Egyptian rousettes contained neutralizing antibodies to CHIKV, and 8/303 (2.6%) contained neutralizing antibodies that were nonspecifically reactive to alphaviruses. These data support the interepidemic circulation of ONNV and CHIKV in Uganda, although Egyptian rousette bats are unlikely to serve as reservoirs for these viruses given the inconsistent occurrence of antibody-positive bats.

## 1. Introduction

Both o’nyong-nyong virus (ONNV) and chikungunya virus (CHIKV) (Family: *Togaviridae,* Genus: *Alphavirus*) are closely related mosquito-transmitted viruses endemic to Uganda [1,2,3,4]. As members of the Semliki Forest virus antigenic complex [5], human infection with either virus is characterized by febrile symptoms, rash, and debilitating polyarthralgia [6,7]. CHIKV was first detected in Uganda in the late 1950s in *Aedes africanus* (Theobald) mosquitoes collected in Zika forest near Entebbe [1,3,8]. This virus had previously been described following an outbreak in Tanzania several years earlier [9,10]. ONNV was first described in Uganda in 1959 [2,4]. Since that time, there have been only episodic outbreaks separated by decades of quiescence [11], while CHIKV has emerged and caused outbreaks globally, particularly over the past twenty years [12,13,14,15,16]. The most recent ONNV outbreak in Uganda, representing the only re-emergence of this virus since 1959, was in the south-central region of the country in 1996–1997, during which an estimated 45% of the at-risk population was infected [11,17,18]. Serological evidence from humans and wildlife support the ongoing interepidemic circulation of both CHIKV and ONNV in East Africa [19,20,21].

While non-human primates likely serve as one reservoir and amplifying host for CHIKV [5,22,23], the wildlife reservoir for ONNV has remained elusive [5]. Owing to evidence that fruit bats are fed upon by mosquitoes in Uganda [24] and that arbovirus seroprevalence among bats in Uganda has been documented [25], we performed additional serological testing on bat serum to look for evidence of sylvatic circulation of ONNV and CHIKV and determine whether or not any of the bat species evaluated could potentially serve as a reservoir for either of these viruses.

## 2. Materials and Methods

### 2.1. Study Location

Bats were captured from multiple locations throughout Uganda during 2011–2013. Specific capture locations, and a map of bat capture and sampling locations are published elsewhere [25,26]. Additional serum samples from Egyptian rousette bats captured from Maramagambo Forest, Queen Elizabeth National Park (QENP) in 2009 [27] were also provided for analysis.

### 2.2. Bat Captures

All bat captures were conducted under the approval of CDC IACUC protocols 1731AMMULX (Division of High Consequence Pathogens and Pathology: Maramagambo samples) and 010-015 (Division of Vector-borne Diseases: all other samples). Bats were captured using harp traps or mist nets, taking appropriate biosafety precautions. Upon capture, bats were placed individually in holding bags. Blood from bats captured in Maramagambo forest was collected and stored as described by Towner et al. [26]. Bats from other locations than Maramagambo forest were treated as follows: bats were anesthetized with halothane and bled by cardiac puncture, then euthanized by halothane overdose and cervical dislocation. Blood was collected directly into serum separator tubes, centrifuged in the field, and placed immediately in liquid nitrogen dry shippers for transport to the laboratory.

### 2.3. Serological Testing

All serum samples were frozen at −80 °C until they were heat inactivated and tested for neutralizing antibodies against CHIKV and ONNV by plaque reduction neutralization test (PRNT) [28]. Alphavirus results were interpreted as follows, due to the unique nature of the one-way antigenic cross-reactivity between CHIKV and ONNV [29]. A CHIKV titer > 4-fold higher than the ONNV titer was considered CHIKV-positive. Any bat with a neutralizing antibody titer for ONNV that was higher than that for CHIKV was considered ONNV-positive, with a minimum ONNV-positive titer as PRNT_80_ = 10, as long as the corresponding CHIKV titer was < 10. Samples with neutralizing antibody titers for CHIKV that were < 4-fold higher than titers for ONNV, or those with equal titers for CHIKV and ONNV were considered non-specific alphavirus-positive. Mouse hyperimmune ascites fluid generated against either CHIKV or ONNV was obtained from the CDC Arbovirus Reference Collection for use as positive control antisera.

## 3. Results

In total, sera from 652 bats were screened for neutralizing antibodies against CHIKV and ONNV. These bats included 400 Egyptian rousette bats (*Rousettus aegyptiacus*), 82 little free-tailed bats (*Chaerephon pumilus*), 15 African straw-colored fruit bats (*Eidolon helvum*), 99 Ethiopian epauletted fruit bats (*Epomophorus labiatus*), 9 Angolan rousette bats (*Myonycteris angolensis*), 33 Angolan mops bats (*Mops condylurus*), 10 Cape long-eared bats (*Nycteris thebaica*), and 4 Noack’s leaf-nosed bats (*Hipposideros ruber*) (Table 1). Forty out of 303 Egyptian rousettes from Maramagambo Forest during 2009, and 1/13 little free-tailed bats from Banga Nakiwogo, Entebbe area during 2011 were ONNV antibody-positive. In addition, 2/303 Egyptian rousettes from Maramagambo forest were antibody-positive for CHIKV, and 8/303 contained neutralizing antibodies that were nonspecifically reactive to alphaviruses (Table 1).

## 4. Discussion

Here, we report exposure of one cave population of Egyptian rousette bats in Uganda sampled during 2009 to both ONNV and CHIKV, and a single little free-tailed bat captured near Entebbe during 2011 to ONNV, determined by the detection of specific neutralizing antibodies in bat serum.

Many reports exist of alphavirus detections, isolations, and seroprevalence in different bat species around the world [30,31], demonstrating that exposure of bats to alphaviruses is not uncommon. In brief, Tonate virus, a strain of Venezuelan equine encephalitis virus, was detected in three bat species in French Guinea [12]. Serological evidence of multiple fruit bat species in Grenada supports exposure to CHIKV [32], infection of mosquitoes experimentally fed on Ross River virus (RRV)-viremic gray-headed flying foxes (*Pteropus pliocephalus*) was possible, although viremia in the bats was low [33], and antibodies reactive to eastern equine encephalitis virus were detected in sera from one great fruit-eating bat (*Artibeus lituratus*) and two Seba’s short-tailed bats (*Carollia perspicillata*) in Trinidad by hemagglutination inhibition [34]. These examples suggest exposure of bats to alphaviruses is occurring, but whether or not bats contribute to the amplification and transmission of these viruses is unknown and likely varies by viral system.

During 2009, approximately 13% of the Egyptian rousette bats in Python cave, Maramagambo forest, had neutralizing antibodies to ONNV, 0.7% had antibodies to CHIKV, and an additional 2.6% had neutralizing antibodies with non-specific alphavirus cross-reactivity based on our criteria. Even though ONNV has not caused an epizootic in Uganda since 1996–1997, the virus may be circulating at low levels during this interepidemic period. Kasokero cave, where some Egyptian rousette bats were sampled, is located in Rakai District where this most recent ONNV outbreak occurred [11,17,18]. However, no evidence for alphavirus exposure was detected in the 53 bats from Kasokero cave during 2013, nor from bats captured from 44 bats Tutum cave in Eastern Uganda during 2012. This inconsistency in seropositivity suggests that Egyptian rousette bats are unlikely reservoir hosts for either CHIKV or ONNV, but rather the population in Maramagambo forest was incidentally exposed to these viruses through blood-feeding mosquitoes. Additional information on the population structure and movement patterns of Egyptian rousette bats in East Africa would also add illuminating perspective on when and where these exposures may have occurred. Whether or not bats shed CHIKV or ONNV through any route that would support direct bat-to-bat transmission has not been investigated.

Assuming exposure occurred through the bite of infected mosquitoes in or near QENP, transmission events could have occurred either at the cave roost location or at a nightly foraging site. ONNV is known to be transmitted by mosquitoes in the genus *Anopheles* [4], and in particular *An. funestus* was implicated in the 1996–1997 outbreak [18]. This mosquito species (*An. funestus* s.s.) is highly anthropophilic and a vector of human malaria in Africa [35], although the blood host preference of *An. funestus* can be variable and include other domestic animals [36]. *Anopheles funestus* s.l. also comprises a large cryptic species complex that includes more exophilic species [37]. The primary vector of CHIKV in more urban settings is *Ae. aegypti*, however this virus can also be transmitted by other anthrophilic *Aedes* species in forested habitats [38,39,40]. In the event these Egyptian rousette bats were foraging at fruit trees in the vicinity of human habitation, exposure to blood-feeding mosquitoes at that location distant from the cave would also be a plausible opportunity for arbovirus exposure. A recent human serosurvey from Uganda confirmed the presence of neutralizing antibodies to CHIKV and/or ONNV in people throughout Uganda [19], including in the Fort Portal area, near QENP.

Alternatively, if the bats were exposed to ONNV in Maramagambo forest, it is unknown what potential mosquito vectors could be responsible, or when this exposure may have occurred. *Anopheles* mosquitoes were not commonly captured in the vicinity of Python cave during the time frame these bats were sampled. Mosquito collections from Maramagambo forest in 2009–2010 yielded over 50 species from 7 genera, of which a very small percentage were anophelines [41]. Mosquitoes that had engorged upon Egyptian rousette bats in Maramagambo forest include *Coquillettidia fuscopennata*, and *Culex decens* group [24]. Both of these mosquito species have documented associations with alphaviruses; both Sindbis virus (SINV) and CHIKV have been isolated from *Cq. fuscopennata*, and Babanki (BBKV) and CHIKV from *Cx. decens* group mosquitoes [41]. Therefore, it is possible that these forest-dwelling species may be responsible for transmission of CHIKV to the Egyptian rousette bats in Python cave. Our serological results also reflect infection with a different alphavirus other than CHIKV or ONNV. We previously detected neutralizing antibodies to BBKV in two wild-caught bats in Uganda from this same sample set, but those BBKV antibody-positive bats comprised one little epauleted fruit bat sampled from Kikaaya in 2011, and one Egyptian rousette bat from Kasokero cave in 2013 [25]. Neither of these two bats had detectable antibody titers against ONNV or CHIKV. Semliki Forest virus (SFV) and SINV are also endemic to Uganda, but the viruses were not included in the test panel for this study.

The detection of such a high neutralizing antibody titer to ONNV in the little free-tailed bat was particularly surprising (Table A1). The antibody titer of the little free-tailed bat was also an order of magnitude greater than all other positive responses in this study and those previously reported [25]. Little free-tailed bats are known to roost in very large numbers in the attics of homes in Uganda, where this one individual bat was captured [42]. It is unknown if this bat was bitten by an infectious mosquito during the day while roosting, or if it would have been orally exposed by consuming an infected mosquito while foraging at night. Limited evidence exists that demonstrate bats can become infected with an arbovirus following consumption of infected mosquitoes. Eastern pipistrelles (*Perimyotis subflavus*) became infected with Japanese encephalitis virus (family: *Flaviviridae*) after consuming infected mosquitoes [43], setting a precedent for this possible route of infection.

Neutralizing antibody titers among the other bats were generally very low (Table A1). Antibody-mediated virus neutralization is not recognized to play a significant role in clearance of Marburg, Ebola, or Sosuga viruses in Egyptian rousette bats [44], which may explain the low titers observed in that species (Table A1). Paweska et al. [45] reported neutralizing antibody titers (PRNT_75_) in Egyptian rousette bats of 1:4 to 1:8, and not all infected bats developed neutralizing antibodies.

## 5. Conclusions

Sometime prior to 2009, ONNV and CHIKV appear to have been circulating in Western Uganda at high enough levels such that approximately 13% of Egyptian rousette bats in Maramagambo forest became exposed and developed detectable neutralizing antibody titers to ONNV and almost 1% to CHIKV. The epidemiological significance of this finding is unknown, but it seems unlikely that these bats could be natural reservoirs for either virus given the lack of seropositivity in the other populations tested. Whether or not Egyptian rousette bats support ONNV or CHIKV replication and could potentially serve as a zoonotic amplifying host during periods of elevated virus circulation is unknown. This evidence for exposure to both CHIKV and ONNV coupled with generic alphavirus antibody detection and prior detections of neutralizing antibodies to BBKV does indicate that Egyptian rousettes are exposed to multiple mosquito-borne alphaviruses. Moreover, a single seropositive little free-tailed bat indicates that this species may be susceptible to ONNV by oral exposure by feeding on infected mosquitoes, but further research is warranted.

## Figures and Tables

**Table 1:** Percentage of bats with significant neutralizing antibody titers (PRNT_80_) against alphaviruses.

			Number (Percent) Positive		
Species	Location	Sample year	ONNV	CHIKV	Alphavirus Nonspecific
Egyptian rousette bat *(Rousettus aegyptiacus)*	Maramagambo forest, QENP *	2009	**40/303 (13%)**	**2/303 (0.7%)**	**8/303 (3%)**
	Tutum Cave, Mt. Elgon	2012	0/44 (0%)	0/44 (0%)	0/44 (0%)
	Kasokero cave, Masaka	2013	0/53 (0%)	0/53 (0%)	0/53 (0%)
African straw-colored fruit bat (*Eidolon helvum*)	Bugonga, Entebbe	2011	0/7 (0%)	0/7 (0%)	0/7 (0%)
	Jinja	2012	0/8 (0%)	0/8 (0%)	0/8 (0%)
Ethiopian epaulletted fruit bat *(Epomphorus labiatus)*	Kikaaya, Kampala	2011	0/7 (0%)	0/7 (0%)	0/25 (0%)
	Buwaya/Kasanje	2011	0/23 (0%)	0/23 (0%)	0/23 (0%)
	Kawuku	2013	0/51 (0%)	0/51 (0%)	0/51 (0%)
Angolan rousette bat *(Myonycteris angolensis)*	Kapkwai Cave, Mt. Elgon	2012	0/9 (0%)	0/9 (0%)	0/9 (0%)
Little free-tailed bat *(Chaerephon pumilus)*	Kisubi/Kawuku	2013	0/69 (0%)	0/69 (0%)	0/69 (0%)
	Banga Nakiwogo	2011	**1/13 (8%)**	0/13 (0%)	0/13 (0%)
Angolan mops bat *(Mops condylurus)*	Banga Nakiwogo	2011	0/33 (0%)	0/33 (0%)	0/33 (0%)
Cape long-eared bat *(Nycteris thebaica)*	Kaptum Cave, Mt. Elgon	2012	0/10 (0%)	0/10 (0%)	0/10 (0%)
Noack’s leaf-nosed bat *(Hipposideros ruber)*	Kapkwai Cave, Mt. Elgon	2012	0/3 (0%)	0/3 (0%)	0/3 (0%)
	Kasokero cave, Masaka	2013	0/1 (0%)	0/1 (0%)	0/1 (0%)

* QENP = Queen Elizabeth National Park; ONNV = o’nyong-nyong virus; CHIKV = chikungunya virus.

## Data Availability

All data have been provided in the manuscript.

## Appendix A

**Table A1:** Endpoint antibody titers of bats seropositive for neutralizing antibodies against o’nyong-nyong virus (ONNV) and chikungunya virus (CHIKV) (PRNT_80_). QENP = Queen Elizabeth National Park, Maramagambo Forest.

				PRNT_80_ Endpoint Titer		
Bat #	Common name	Location	Date	CHIKV	ONNV	Results Reported
182	Little free-tailed bat	Banga Nakiwogo	2011	<10	≥320	ONNV
1223	Egyptian rousette	QENP	2009	<10	10	ONNV
1225	Egyptian rousette	QENP	2009	<10	20	ONNV
1229	Egyptian rousette	QENP	2009	<10	10	ONNV
1264	Egyptian rousette	QENP	2009	<10	10	ONNV
1305	Egyptian rousette	QENP	2009	10	40	ONNV
1306	Egyptian rousette	QENP	2009	<10	20	ONNV
1313	Egyptian rousette	QENP	2009	10	40	ONNV
1314	Egyptian rousette	QENP	2009	<10	20	ONNV
1316	Egyptian rousette	QENP	2009	<10	20	ONNV
1318	Egyptian rousette	QENP	2009	<10	20	ONNV
1324	Egyptian rousette	QENP	2009	<10	40	ONNV
1334	Egyptian rousette	QENP	2009	<10	20	ONNV
1348	Egyptian rousette	QENP	2009	10	10	Alphavirus
1354	Egyptian rousette	QENP	2009	<10	20	ONNV
1363	Egyptian rousette	QENP	2009	<10	20	ONNV
1367	Egyptian rousette	QENP	2009	10	20	ONNV
1375	Egyptian rousette	QENP	2009	10	<10	Alphavirus
1382	Egyptian rousette	QENP	2009	20	<10	CHIKV
1385	Egyptian rousette	QENP	2009	10	80	ONNV
1386	Egyptian rousette	QENP	2009	<10	20	ONNV
1387	Egyptian rousette	QENP	2009	<10	20	ONNV
1388	Egyptian rousette	QENP	2009	<10	10	ONNV
1390	Egyptian rousette	QENP	2009	10	<10	Alphavirus
1391	Egyptian rousette	QENP	2009	20	<10	CHIKV
1392	Egyptian rousette	QENP	2009	10	<20	Alphavirus
1406	Egyptian rousette	QENP	2009	10	<10	Alphavirus
1455	Egyptian rousette	QENP	2009	<10	10	ONNV
1456	Egyptian rousette	QENP	2009	<10	40	ONNV
1467	Egyptian rousette	QENP	2009	<10	20	ONNV
1469	Egyptian rousette	QENP	2009	<10	20	ONNV
1471	Egyptian rousette	QENP	2009	<10	10	ONNV
1475	Egyptian rousette	QENP	2009	<10	10	ONNV
1478	Egyptian rousette	QENP	2009	<10	40	ONNV
1480	Egyptian rousette	QENP	2009	<10	20	ONNV
1483	Egyptian rousette	QENP	2009	<10	40	ONNV
1484	Egyptian rousette	QENP	2009	10	<10	Alphavirus
1486	Egyptian rousette	QENP	2009	<10	40	ONNV
1516	Egyptian rousette	QENP	2009	<10	10	ONNV
1518	Egyptian rousette	QENP	2009	10	<10	Alphavirus
1539	Egyptian rousette	QENP	2009	<10	20	ONNV
1543	Egyptian rousette	QENP	2009	<10	20	ONNV
1546	Egyptian rousette	QENP	2009	<10	40	ONNV
1560	Egyptian rousette	QENP	2009	40	20	Alphavirus
1571	Egyptian rousette	QENP	2009	<10	20	ONNV
1572	Egyptian rousette	QENP	2009	<10	20	ONNV
1588	Egyptian rousette	QENP	2009	<10	10	ONNV
1600	Egyptian rousette	QENP	2009	<10	20	ONNV
1601	Egyptian rousette	QENP	2009	10	20	ONNV
1608	Egyptian rousette	QENP	2009	<10	20	ONNV
1617	Egyptian rousette	QENP	2009	<10	20	ONNV
